# “Mind the Gap”: Hi-C Technology Boosts Contiguity of the Globe Artichoke Genome in Low-Recombination Regions

**DOI:** 10.1534/g3.120.401446

**Published:** 2020-08-18

**Authors:** Alberto Acquadro, Ezio Portis, Danila Valentino, Lorenzo Barchi, Sergio Lanteri

**Affiliations:** DISAFA Plant Genetics & Breeding, University of Torino, Largo Paolo Braccini 2, 10095, Grugliasco (Torino) Italy

**Keywords:** Genomics, NGS, HI-C libraries, *Cynara cardunculus*

## Abstract

Globe artichoke (*Cynara cardunculus* var. *scolymus*; 2n2x=34) is cropped largely in the Mediterranean region, being Italy the leading world producer; however, over time, its cultivation has spread to the Americas and China. In 2016, we released the first (v1.0) globe artichoke genome sequence (http://www.artichokegenome.unito.it/). Its assembly was generated using ∼133-fold Illumina sequencing data, covering 725 of the 1,084 Mb genome, of which 526 Mb (73%) were anchored to 17 chromosomal pseudomolecules. Based on v1.0 sequencing data, we generated a new genome assembly (v2.0), obtained from a Hi-C (Dovetail) genomic library, and which improves the scaffold N_50_ from 126 kb to 44.8 Mb (∼356-fold increase) and N_90_ from 29 kb to 17.8 Mb (∼685-fold increase). While the L_90_ of the v1.0 sequence included 6,123 scaffolds, the new v2.0 just 15 super-scaffolds, a number close to the haploid chromosome number of the species. The newly generated super-scaffolds were assigned to pseudomolecules using reciprocal blast procedures. The cumulative size of unplaced scaffolds in v2.0 was reduced of 165 Mb, increasing to 94% the anchored genome sequence. The marked improvement is mainly attributable to the ability of the proximity ligation-based approach to deal with both heterochromatic (*e.g.*: peri-centromeric) and euchromatic regions during the assembly procedure, which allowed to physically locate low recombination regions. The new high-quality reference genome enhances the taxonomic breadth of the data available for comparative plant genomics and led to a new accurate gene prediction (28,632 genes), thus promoting the map-based cloning of economically important genes.

Globe artichoke (*Cynara cardunculus* var. *scolymus*) is native to the Mediterranean region, where it is largely cropped for the production of edible immature inflorescences, being Italy the leading world producer (about 388K tons in 2017) (FAO). Immigrants introduced this crop to the Americas, and more recently its cultivation has spread to the eastern part of the world (*e.g.*, China). *C. cardunculus* includes two further taxa: the cultivated cardoon (var. *altilis*), grown for the production of fleshy stems (Portis *et al.* 2005a), and wild cardoon (var. *sylvetris*), the progenitor of both cultivated forms (Portis *et al.* 2005b; Mauro *et al.* 2009). The three taxa are exploited for the production of a number of nutraceutically and pharmaceutically active compounds such as phenylpropanoids (Pandino *et al.* 2015) and sesquiterpene lactones (cynaropicrin and grosheimin) (Eljounaidi *et al.* 2014) and particularly cultivated cardoon is a source of both ligno-cellulosic biomass and seed oil for edible and biofuel uses (Portis *et al.* 2018).

The continuous evolution of Next Generation Sequencing (NGS) technologies is triggering data production, and analysis, and massively parallel sequencing has proven revolutionary, shifting the paradigm of genomics to address biological questions at a genome-wide scale (Koboldt *et al.* 2013). Today, in the case of relatively small genomes (*e.g.*, bacterial or viral), complete genome sequences can frequently be reconstructed computationally; however, the reconstruction of large and complex eukaryotic genomes, such as the ones of plants, continue to pose significant challenges (Ghurye and Pop 2019). Short reads technology (*e.g.*: Illumina) is generally combined with long-reads sequencing technologies, such as Single-molecule real-time sequencing (SMRT, Pacific Biosciences) or nanopore sequencing (Oxford Nanopore technologies). Furthermore, with the goal of improving the assembly quality, cutting edge scaffolding technologies such as linked-reads (10X Genomics), optical mapping (Bionano Genomics) and proximity ligation methods (Hi-C, Dovetail Genomics) are adopted.

Hi-C is a proximity ligation based method, which relies on the fact that, after fixation, segments of DNA in close proximity in the nucleus are more likely ligated together and sequenced as pairs in respect to more distant regions. As a result, the number of read pairs between intra-chromosomal regions is a slowly decreasing function of the genomic distance between them. Furthermore, Hi-C could theoretically allow score contact frequency between virtually any pair of genomic loci (Lieberman-Aiden *et al.* 2009).

Globe artichoke harbors a highly heterozygous genetic background, which hampers the production of a reference assembly. We developed an inbred genotype with a 10% of residual heterozygosity, of which we released the first globe artichoke genome sequence (Scaglione *et al.* 2016). The assembly (v1.0) was generated using ∼133-fold Illumina sequencing data and covered 725 of the 1,084 Mb genome. Through genetic mapping, we anchored 526 Mb (73%) of the genome sequence to 17 chromosomal pseudomolecules, although ∼199 Mb (27%) remained unplaced. More recently, we released an improved annotation (v1.1) of the v1.0 assembly and the genome sequence of four globe artichoke genotypes (Acquadro *et al.* 2017), as well as a genotype of cultivated cardoon.

Here we report on a new reference genome (v2.0), obtained by sequencing a Hi-C genomic library and assembling data with previously generated sequence datasets. This new chromosome-level version is characterized by a high contiguity and reduces drastically the number of unplaced scaffolds.

## Materials and methods

### Hi-C Library preparation, sequencing and assembling

Fresh etiolated leaves of a globe artichoke inbred line (2C), from which we generated the reference genome (Scaglione *et al.* 2016), was provided to Dovetail Genomics (https://dovetailgenomics.com). DNA was extracted from leaf samples and used to construct a Hi-C library following manufacturer protocols (Putnam *et al.* 2016). The Hi-C library was then quality checked through sequencing (2M PE 75bp reads, Illumina, MiSeq) and reads mapped back to the draft assembly. Afterward, extensive Illumina sequencing was performed with an Illumina HiSeq X instrument (PE150bp reads chemistry).

Hi-C data, as well as 20-30X shotgun data (project PRJNA238069), were used in the HiRise pipeline (https://github.com/DovetailGenomics/HiRise_July2015_GR) to perform scaffolding of the input assembly (v1.0), adopting standard procedures. BlastN was used to reconcile superscaffolds with pseudomolecule nomenclature (Scaglione *et al.* 2016).

### Gene prediction

The new assembly was masked using RepeatMasker (Smit *et al*. 2013–2015) using a combination of homology-based and *de novo* approaches. After a soft masking step, a gene prediction was performed using Maker-P (Campbell *et al.* 2014). Augustus (Stanke *et al.* 2006) Hidden Markov Models and SNAP (Bromberg and Rost 2007) gene prediction algorithms were combined with artichoke transcripts available in NCBI and proteins alignments as evidence to support prediction. All predicted gene models were filtered to maintain only those with a AED ≤ 0.35; this value measures the concordance between the predicted model and the experimental tests, with reliability of the higher models and low AED values. For each predicted gene, the gene function was assigned by a BlastP (Altschul *et al.* 1990) search against the Uniprot/Swissprot Viridiplantae database (The UniProt Consortium 2014), using the default parameters, with the exception of the e-value (< 1e^-5^). The sequences of the predicted proteins were also noted using InterproScan (v. 5.33-72.0; (Jones *et al.* 2014)) compared to all the available databases (ProSitePro 2018_02 (Sigrist *et al.* 2013), PANTHER-12 (Mi *et al.* 2013), Coils-2.2.1 (Lupas *et al.* 1991), PIRSF-3.02 (Wu *et al.* 2004), Hamap-2018_3 (Lima *et al.* 2009), Pfam-32 (Punta *et al.* 2012), ProSitePatterns 2018_02 (Sigrist *et al.* 2013), SUPERFAMILY-1.75 (de Lima Morais *et al.* 2011), ProDom-2006.1 (Bru *et al.* 2005), SMART-7.1 (Letunic *et al.* 2012), Gene3D-4.2 (Lees *et al.* 2012) and TIGRFAM-15 (Haft *et al.* 2013)).

The MIReNA (Mathelier and Carbone 2010) software was used for the identification of high confidence miRNA-coding sequences (miRBase release 21 (Kozomara and Griffiths-Jones 2011): high confidence database). An homology search was conducted with known miRNAs from an array of 13 species (plants and algae), including: *Solanum lycopersicum*, *Solanum tuberosum*, *Nicotiana tabacum*, *Vitis vinifera*, *Arabidopsis thaliana*, *Oryza sativa*, *Populus trichocarpa*, *Medicago trunculata*, *Zea mays*, *Picea abies*, *Triticum aestivum*, *Physcomitrella patens*, *Chlamydomonas reinhardtii*. MIReNA was run with default parameters and the maximum number of allowed mismatches between known miRNAs and putative miRNAs was set to 10.

### Genome integrity and completeness

The QUAST pipeline (Mikheenko *et al.* 2018), which includes the BUSCO software (Simão *et al.* 2015), was used for the comparison among the new and the previous versions of the genome. Plant dataset (Embryophyta, odb9) was downloaded from Busco (Simão *et al.* 2015) and manually implemented in the QUAST pipeline. A comparison between different versions of the globe artichoke assembled genomes was conducted retrieving co-linear blocks through Last aligner (Kiełbasa *et al.* 2011). Only blocks with pairwise minimal identity major/equal than 99% were plotted using Circos tool (Krzywinski *et al.* 2009).

### Data availability

Raw reads are publicly available in the NCBI sequence read archive under the bioproject: PRJNA238069. The reference assembly (v2.0) and annotation data are either available for downloading from http://www.artichokegenome.unito.it.

## Results and discussion

### Sequencing, assembling and metrics

We developed a new genome assembly (v2.0) using Hi-C technology, which is based on proximity ligation and massively parallel sequencing to probe the three-dimensional structure of chromosomes within the nucleus, and capture interactions by paired-end sequencing (Putnam *et al.* 2016; Ghurye *et al.* 2017). A single genomic library was sequenced using Illumina chemistry and a total of 156,683,926 pair end reads (2x150bp; 47.01 Gbp) generated. Hi-C reads were used in the assembly procedure, by adopting the existing genomic scaffolds as starting sequences (Scaglione *et al.* 2016), through the HiRise assembly pipeline, and enabled an accurate assembly of the globe artichoke genome up to the chromosome-level (Table 1). In all 5,023 super-scaffolds were generated, with an average size of 144,578 bp. The largest 18 super-scaffolds were assigned to chromosomes using reciprocal blast procedures. The 17 pseudomolecules were reconstructed also by joining together two super-scaffolds (13,663 and 1,119) in chromosome 6.

**Table 1:** – Metrics for the v1.0 (reference) scaffolds, the v1.0 (reference) pseudomolecules, and v2.0 (Hi-C) super-scaffolds

Metrics	v2.0 (Hi-C)	v1.0 (pseudomolecules)	v1.0 (scaffolds)
Total assembly size	726,213,971	725,337,666	725,334,175
Number of contigs/scaffolds	5,023	8,344	13,662
Average size	144,578	86,929	53,091
N_50_	44,809,927	25,947,084	125,836
L_50_	7	9	1,411
N_75_	31,669,976	166,465	59,381
L_75_	11	98	3,545
N_90_	23,740,492	45,160	31,081
L_90_	15	1,384	5,853
Busco, complete genes (%)	89.65	89.44	89.44
Busco, partial genes (%)	3.06	1.98	1.98
Busco, overall (%)	92.71	91.42	91.42

**Figure 1:**
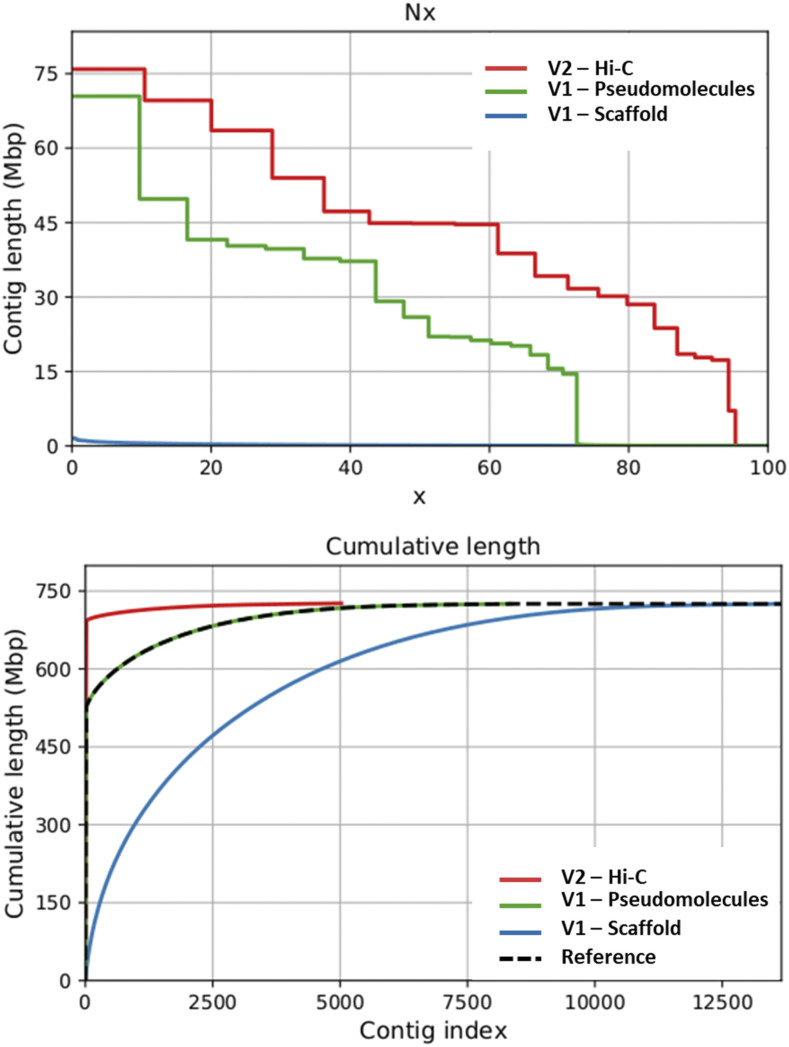
- Contiguity improvement performed on v1.0 genome (scaffolds), v1.0 reference genome (pseudomolecules plus unplaced scaffolds) and v2.0 genome (Hi-C superscaffolds). Top picture: N_x_ statistics with x varying between 1 and 100. Bottom picture: it represents the cumulative length increment of the genome through the scaffold/contig addition.

To assess the improvement obtained in the new assembly, a first comparison was performed between the Hi-C pseudomolecules (v2.0) toward the original scaffolds of v1.0. This resulted in an improvement of the N_50_ value, which increased from 126 kb to 44.8 Mb (∼356-fold increase) and the N_90_, which reached 17.8 Mb compared to the original v1.0 value of 29 kb (∼685-fold increase). The huge improvement of the HI-C assembly was also highlighted by the L_90_ value, which dramatically drop down from 6,123 scaffolds in the v1.0 version to just 15 super-scaffolds, a number close to the haploid chromosome number of the species. Similar remarkable improvements were also highlighted by comparing the Hi-C superscaffolds with the anchored version of the genome (v1.0, pseudomolecules-based plus scaffolds) (Figure 1; Table 1). As an example, the N_50_ value jumped from ∼26Mb in v1.0 to ∼45Mb in v2.0, while the L_90_ dropped down from 1,384 of the V1.0 to 15 in the HI-C assembly.

**Figure 2:**
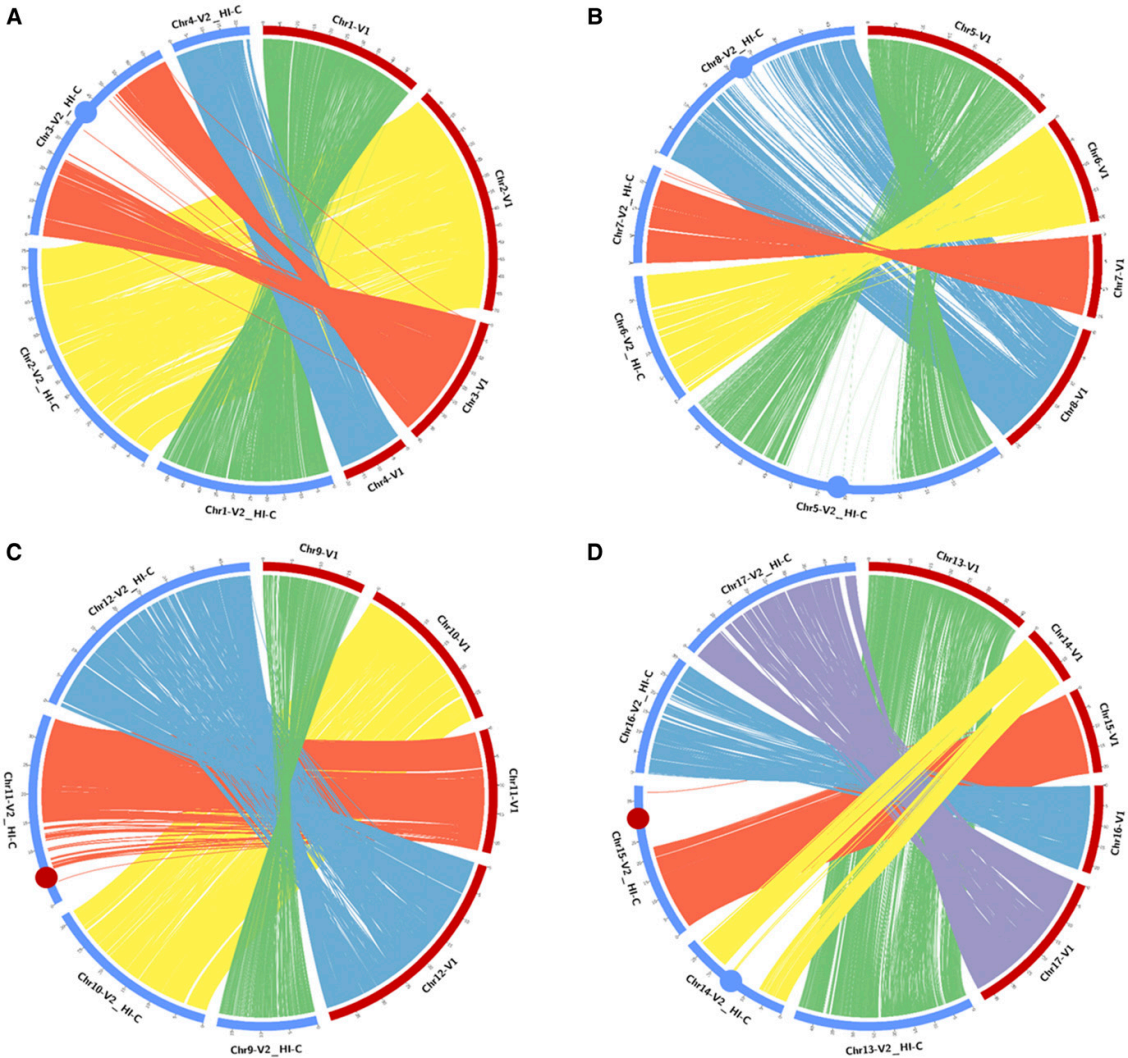
Circos plot depicting the syntenic relationships between the chromosomes of the globe artichoke genome (v1.0, pseudomolecules, in red) and the new assembly (v2.0, Hi-C superscaffold, in blue). A - from chromosome 1 to 4; B - from chromosome 5 to 8; C) from chromosome 9 to 12; D) from chromosome 13 to 17. Blue dots highlights extended regions in the v2.0 assembly in pericentromeric positions in metacentric/sub-metacentric chromosomes. Red dots highlights extended regions in the v2.0 assembly in pericentromeric positions in acrocentric/telocentric chromosomes.

Focusing on the unanchored portion of the genome (namely Chr0), the ∼199 Mb of unplaced sequence in v1.0, which included 8,327 scaffolds, was decreased to less than ∼34 Mb (5,005 sequences), as ∼165 Mb (∼83%) were assigned to super-scaffolds. On the whole, the percentage of anchored genome increased to ∼94% and the chromosome size extended with a medium gain of ∼36% (Table 4). The highest increase was observed in chromosome 14, whose size enlarged of ∼14Mb (97%), in respect to the v1.0. Some chromosomes showed scattered insertion of the new anchored scaffolds (*i.e.*: 1, 2, 6, 9, 10, 12, 13), while in others (*i.e.*: 3, 4, 5, 7, 8, 11, 14, 15, 16, 17) distinct extensive regions (ranging from 2.9Mb to 29.3Mb) were anchored (Figure 2).

### Genome annotation

In the genome Hi-C version, the annotation pipeline predicted 28,632 genes, a higher number than the one predicted in v1.0 (*i.e.*: 26,889; (Scaglione *et al.* 2016)), and very close to the one we recently obtained following the genome reconstruction of globe artichoke genotypes (*i.e.*: 28,310, v1.1) (Acquadro *et al.* 2017). The number of genes in unplaced scaffolds was just 557 (1,9% of the total genes), raising up the number of genes (+4,180, 17%) placed on pseudomolecules. This number (557) is by far lower than the one located on Chr0 in the two previous structural annotations: *i.e.*, 2,994 (Scaglione *et al.* 2016) and 3,471 (Acquadro *et al.* 2017). Following Busco (Simão *et al.* 2015) analysis, as expected the number of represented orthologs in Hi-C assembly (92.7%) was just slightly higher compared to the previous version (91.4%), being essentially unaltered the sequences of the contigs during the assembly process (data not shown).

The InterProScan analyses highlighted about 80% of the predicted proteins with at least one IPR domain, in line with the previous v1.0 and v1.1 annotation. Among the top 20 SUPERFAMILY domains, listed in Table 2, the most abundant in all the genomes was SSF52540 (P-loop containing nucleoside triphosphate hydrolase), which is involved in several UniPathways, including chlorophyll or coenzyme A biosynthesis. The other most abundant Superfamilies were: SSF56112 (protein Kinase-like domain), which acts on signaling and regulatory processes in the eukaryotic cell, SSF52058 (Leucine-rich repeat domain, L domain-like), which is related to resistance to pathogens and SSF48371 (Armadillo-type fold), which plays a role in defense response and translation factor activity. These findings are comparable to both v1.0 and v1.1 annotations, suggesting that Hi-C had a greater effect in improving the quality of the genome sequence than its annotation.

**Table 2:** - TOP20 Superfamily in the v2 annotation, after Interproscan5 analyses and compared to v1 and v1.1 annotations

Domain	Description	v2	v1.1	v1.0
SSF52540	P-loop containing nucleoside triphosphate hydrolases	1,346	1,347	1,311
SSF56112	Protein kinase-like (PK-like)	1,310	1,309	1,303
SSF52058	L domain-like	757	806	772
SSF57850	RING/U-box	530	530	529
SSF48371	ARM repeat	491	493	481
SSF51735	NAD(P)-binding Rossmann-fold domains	441	443	427
SSF48452	TPR-like	404	402	408
SSF54928	RNA-binding domain, RBD	431	417	401
SSF53474	alpha/beta-Hydrolases	390	397	391
SSF48264	Cytochrome P450	370	380	373
SSF46689	Homeodomain-like	372	366	372
SSF52047	RNI-like	292	295	296
SSF53335	S-adenosyl-L-methionine-dependent methyltransferases	288	288	289
SSF50978	WD40 repeat-like	278	281	281
SSF52833	Thioredoxin-like	271	272	275
SSF53756	UDP-Glycosyltransferase/glycogen phosphorylase	250	251	241
SSF81383	F-box domain	240	238	241
SSF49503	Cupredoxins	226	230	241
SSF51445	(Trans)glycosidases	235	238	241

From a search against miRBase 21 high confidence database, species-specific miRNAs were predicted. The total number of predicted non-redundant was 144 (in 253 genome regions of the reference 2C), in line with what previously reported on annotation v1.1 (143 (Acquadro *et al.* 2017). The identified miRNAs belong to 37 families (Table 3), slightly lower than the ones previously reported (Acquadro *et al.* 2017). Notwithstanding, the most highly-represented miRNA families are shared between the two annotations, which are conserved in many taxonomic groups, as already spotted in previous studies (Cuperus *et al.* 2011; Chávez Montes *et al.* 2014; Barchi *et al.* 2019).

**Table 3:** - miRNA families in the v2.0 annotation compared to v1.1 annotation

miRNA family	Annotation v2.0	Annotation v1.1
156	14	15
7699	13	14
166	18	13
172	7	9
399	10	8
396	8	7
169	10	6
393	3	6
160	4	5
164	3	5
171	8	5
167	3	3
168	4	3
319	9	3
394	3	3
159	3	2
390	1	2
403	2	2
444	1	2
479	0	2
1030	0	2
1446	1	2
2630	3	2
157	1	1
397	1	1
398	1	1
408	0	1
530	1	1
824	0	1
837	1	1
902	0	1
1155	1	1
2079	0	1
2651	1	1
2657	0	1
2658	1	1
2673	0	1
2680	0	1
3633	0	1
4414	1	1
5254	1	1
5258	1	1
5559	0	1
5751	0	1
7696	1	1
1040	1	0
1044	1	0
5237	1	0
6463	1	0

### Mis-assembly level and co-linearity among assemblies

The Hi-C increased of about 30% the size of anchored genome, and accordingly the majority of the newly assembled chromosomes increased their size (Table 4). In particular, chromosomes 3, 5, 8, 11, 14 and 15 expanded of at least 50% in size, compared to the v1.0. (Figure 2). The Quast (Gurevich *et al.* 2013) analysis highlighted that 4,727 scaffolds were mis-assembled. The mis-assemblies were grouped in 3,553 re-locations on the same pseudomolecule, 1,157 translocations and 17 inversions. Following a more in-depth analysis, the mis-assembled scaffolds corresponded to just 54.6Mb of genomic sequence, which included small size fragments (average ∼11.6Kb, median ∼6.1Kb). Relocation involved ∼41.9 Mb (average ∼11.8Kb, median ∼6.6 Kb). Inversions involved ∼0.2 Mb (average ∼12.1 Kb, median ∼11.9 Kb). Translocations involved ∼12.4 Mb (average ∼10.8 Kb, median ∼4.3 Kb).

**Table 4:** - Comparison in length between v1.0 (reference) pseudomolecules and v2.0 (Hi-C) super-scaffolds. Number of genes predicted from v1.0 and v2.0 are shown and compared. The number of genes reported in Acquadro *et al.* (2017) (annotation v1.1) predicted on the v1.0 assembly are also shown

	Size assembly (bp)				N° Genes			
Chromosome	v2.0	v1.0	Δ (bp)	Ratio (%) v2.0/v1.0	v2.0	v1.1	v1.0	Ratio (%) v2.0/v1.0
**1**	53,988,940	49,754,839	4,234,101	9%	2,881	2,692	2,630	10%
**2**	75,886,343	70,441,430	5,444,913	8%	2,696	2,502	2,351	15%
**3**	69,604,505	40,297,365	29,307,140	73%	2,261	1,942	1,868	21%
**4**	23,740,492	20,164,318	3,576,174	18%	1,104	991	962	15%
**5**	63,544,927	37,196,517	26,348,410	71%	1,967	1,723	1,640	20%
**6**	24,383,717	20,634,051	3,749,666	18%	1,084	956	903	20%
**7**	18,502,611	15,568,887	2,933,724	19%	1,003	933	907	11%
**8**	44,609,785	25,947,084	18,662,701	72%	1,529	1,250	1,196	28%
**9**	17,815,532	18,344,014	−528,482	−3%	1,061	1,047	1,006	5%
**10**	31,669,976	29,133,143	2,536,833	9%	1,609	1,516	1,436	12%
**11**	34,212,861	22,016,825	12,196,036	55%	1,611	1,459	1,453	11%
**12**	44,809,927	39,693,055	5,116,872	13%	1,590	1,473	1,404	13%
**13**	44,877,405	41,551,399	3,326,006	8%	2,077	1,873	1,801	15%
**14**	28,499,371	14,487,748	14,011,623	97%	1,003	669	646	55%
**15**	38,772,909	21,275,025	17,497,884	82%	1,751	1,501	1,466	19%
**16**	30,156,653	21,933,510	8,223,143	37%	1,193	964	949	26%
**17**	47,245,614	37,737,787	9,507,827	25%	1,655	1,349	1,277	30%
**Unplaced scaffold**	33,892,403	199,160,669	−165,268,266	−83%	557	3,470	2,994	−81%
**Chromosomes**	692,321,568	526,176,997	+166,144,571	32%	28,075	24,840	23,895	17%
**Total assembled**	**726,213,971**	**725,337,666**	**876,305**	**0.12%**	**28,632**	**28,310**	**26,889**	**6%**

The Hi-C and the v1.0 of the globe artichoke genome assembly were highly co-linear (pseudomolecules plus un-placed scaffold; Figure 2). The remarkable improvement in size of the Hi-C assembly is attributable to the ability of the proximity ligation-based approach to deal with heterochromatic (pericentromeric and telomeric) regions. The latter are characterized by a low recombination rate, low gene density and high TE accumulation (Nachman 2002), thus their analysis is a tough task (Zhang *et al.* 2014) when a classical genetic mapping approach relying on the recombination rate (Scaglione *et al.* 2016) is used. This is the case of v1.0. genome assembly, while the v2.0 was based on the proximity ligation technology, which is recombination rate aware. The case of chromosomes 3, 5, 8, 14 is emblematic. A clear un-aligned region (“extended gap”) was present in their metacentric/sub-metacentric region in version 1.0, which in chromosomes 3 and 5 spanned up to 30Mbs. Similarly, in the terminal region of chromosomes 11 and 15, which in a previous study (Scaglione *et al.* 2016) appeared to be telocentric/acrocentric on the basis of their gene frequency, some scaffolds were missing in v1.0, but correctly assigned in v2.0.

**Figure 3:**
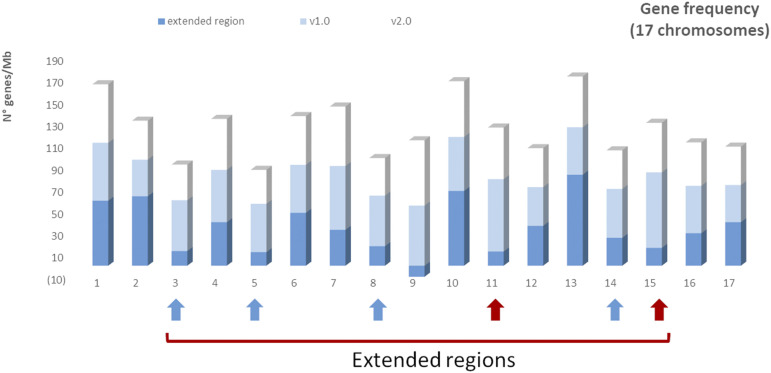
Gene frequency expressed in n° of genes/Mb calculated at chromosome level for the v1.0 genome (light blue bars), v2.0 genome (white bars) and newly extended regions. Blue arrows show newly extended regions in the v2.0 assembly in pericentromeric positions in metacentric/sub-metacentric-like chromosomes. Red arrows highlights newly extended regions in the v2.0 assembly in pericentromeric positions in acrocentric/telocentric-like chromosomes.

All this is confirmed by the fact that the gene frequency of the newly placed scaffolds in the v2.0 assembly was just 29 genes/Mb, by far lower than the average gene frequency detected in both v1.0 and v2.0 (45 genes/Mb), and that the large newly extended regions in chr. 3, 5, 8, 11, 14 and 15 showed a furtherly reduced gene frequency (16 genes/Mb, see Figure 3).
